# Microbiota-ear-brain interaction is associated with generalized anxiety disorder through activation of inflammatory cytokine responses

**DOI:** 10.3389/fimmu.2023.1117726

**Published:** 2023-03-09

**Authors:** Renyi Feng, Qingyong Zhu, Qingchen Li, Yanping Zhai, Jiuqi Wang, Chi Qin, Dongxiao Liang, Rui Zhang, Haiyan Tian, Han Liu, Yongkang Chen, Yu Fu, Xuejing Wang, Xuebing Ding

**Affiliations:** ^1^ Department of Neurology, The First Affiliated Hospital of Zhengzhou University, Zhengzhou, Henan, China; ^2^ Institute of Parkinson and Movement Disorder, Zhengzhou University, Zhengzhou, Henan, China

**Keywords:** generalized anxiety disorder, ear canal microbiome, microbiota-ear-brain interaction, inflammatory response, anxiety

## Abstract

**Introduction:**

Generalized anxiety disorder (GAD) is one of the most enduring anxiety disorders, being associated with increased systemic inflammation. However, the trigger and mechanisms underlying the activation of inflammatory cytokine responses in GAD remain poorly understood.

**Materials and methods:**

We characterized the ear canal microbiome in GAD patients through 16S rRNA gene sequencing and metagenomic sequencing and identified the serum inflammatory markers in GAD patients. Spearman correlations were applied to test the relationship between the microbiota changes and systemic inflammation.

**Results:**

Our findings showed the higher microbial diversity, accompanied with the significantly increased abundance of Proteobacteria, and decreased abundance of Firmicutes in the ear canal of GAD participants compared to that of the age- and sex-matched healthy controls (HC). Metagenomic sequencing showed that Pseudomonas aeruginosa were significantly increased at species-level in GAD patients. Furthermore, we observed the relative abundance of Pseudomonas aeruginosa was positively associated with elevated systemic inflammatory markers and the severity of disease, suggesting that these ear canal microbiota alterations might be correlated with GAD by activating the inflammatory response.

**Conclusions:**

These findings indicate that microbiota-ear-brain interaction via upregulating inflammatory reaction involve in the development of GAD, as well as suggest that ear canal bacterial communities may be a target for therapeutic intervention.

## 1 Introduction

Generalized anxiety disorder (GAD) is the most common anxiety disorder, affecting approximately 4% to 7% of the worldwide population and the incidence is increasing worldwide (1, 2). As a chronic debilitating psychiatric disorder, GAD imposes a large burden on patients, impairing their quality of life as well as their ability to work and function socially (3, 4). In addition, GAD is often comorbid with other mental disorders, especially depression, as well as with somatic disorders such as autonomic dysfunction and gastrointestinal, cardiovascular, respiratory and neurological disorders (5–7).

Increasing evidence suggests an involvement of inflammatory pathways in the pathogenesis of anxiety disorder (8). Proinflammatory changes are linked to dysregulate the hypothalamus-pituitary-adrenal (HPA) axis responses caused by chronic stress as represented by persistent anxiety states, thereby increasing the risk for excessive systemic inflammation (9). Studies of GAD patients have reported increased levels of pro-inflammatory cytokines and decreased levels of anti-inflammatory cytokines compared to healthy individuals (10, 11). Meanwhile, serum levels of inflammatory cytokines were significant positive correlations between anxiety severity (12). Several reports show that exposure to pathogenic infection of *Campylobacter jejuni* or *Trichuris muris* caused colonic inflammation and increased anxiety-like behavior (13, 14). Additionally, antibiotic and probiotic treatment can reverse inflammation-related increased anxiety-like behavior (15–17). The studies described above suggest that increased inflammation is associated with increased anxiety-like behavior.

A more recent study provided evidence that the microbiome has emerged as a key environmental factor affecting human health and disease, especially mental disorders (18, 19). Previous studies have demonstrated that the gut microbiome plays a central role in anxiety disorders through microbiota-gut-brain axis (15, 20). Fecal microbiota of patients suffering from with GAD showed decrease of short-chain fatty acid (SCFA)-producing bacteria and overgrowth of bacteria including *Escherichia-Shigella*, *Fusobacterium* and *Ruminococcus gnavus* (21). Reduced SCFA production in GAD patients could result in intestinal barrier dysfunction that could compromise proper immune responses and ultimately contribute to brain dysfunction (22). Not only gut microbiome composition, oral microbiome composition and serum microbiome composition were also associated with anxiety symptoms (23–25).

In the present pilot work, we characterized the composition of the ear canal microbiome in GAD patients compared to healthy controls (HC). Moreover, we investigated the relationship among the microbiota changes, systemic inflammatory markers and the severity of GAD in GAD patients. Our trial provides new insights into the role of microbiota-ear-brain interaction in the pathogenesis of GAD by upregulating inflammatory reaction.

## 2 Materials and methods

### 2.1 Study design and patient recruitment

A total of 47 GAD patients and 33 HC were recruited from the Department of Neurology of The First Affiliated Hospital of Zhengzhou University. All participants provided written informed consent, and ethics approval was obtained from the Ethics Committee of The First Affiliated Hospital of Zhengzhou University (2022-KY-0851-002). GAD patients were defined by two experienced neurologists and psychiatrist in our department who arrived at a consistent conclusion of the disease, meeting the diagnostic criteria of GAD according to the Structured Clinical Interview for the Diagnostic and Statistical Manual of Mental Disorders (DSM-V) (26). Exclusion criteria were: (i) with severe major depression, bipolar disorder, panic disorder, psychotic disorders, posttraumatic stress disorder, substance abuse/dependence, and significant suicidal ideation etc.; (ii) complicated by heart, lung, kidney, liver, blood, autoimmune diseases, and malignant tumors; (iii) complicated by cerebral vascular disease, neurodegenerative diseases or other diseases that may influence the structure and function of the brain; (iv) immunosuppressive agents and antibiotic use within 3 months prior to sample collection. Participants were not taking psychotropic medication for within 4 weeks or were taking a stable dose for at least 6 weeks. In addition, 33 age and sex-matched controls were enrolled from spouses or accompanied friends of the GAD patients, which had similar lifestyles and the same geographic area. For healthy controls, exclusion criteria included current or past psychiatric disorders and psychoactive medications; history of systemic diseases or co-morbidities; antibiotic use within 3 months before sample collection; and known active bacterial, fungal, parasitic, or viral infections.

### 2.2 Sample collection and clinical data collection

Bilateral ear swabs were taken from subjects enrolled in the study on the second day after admission. The external ear canal depths average 27 mm and range from 20 to 34 mm (27), a 20-mm safe bar was used to ensure safety. Ear canal samples were collected by sterile swabs, inserted into the ear canal as close to the tympanic membrane as the individuals would permit and rotating 180° for 5 s. The swabs were stored without storage media (dry) and placed directly into the sterile container, transferred on ice, and stored at -80°C within 2 h. We used the 14-item Hamilton Anxiety Scale (HAMA) and Self-Rating Anxiety Scale (SAS) for assessing subjective symptoms. The Pittsburgh sleep quality index (PSQI), Composite Autonomic Symptom Score (COMPASS 31), Gastrointestinal Symptom Rating Scale (GSRS), Fatigue Severity Scale (FSS), Dizziness Handicap Inventory (DHI) were used to assess the nonspecific physical and psychological symptoms.

Venous blood samples were collected from all participants using 10 ml test tubes and centrifuged at 3000 rpm at 4°C for 10 min. The supernatant was collected for serum cytokines analysis. The serum was stored at -80°C until all participants were collected. Serum levels of pro-inflammatory cytokines [interleukin (IL)-6, interferon (IFN)-γ, tumor necrosis factor-alpha (TNF-α), IL-1β, lipopolysaccharide (LPS) and LPS-binding protein (LBP)] levels were measured using an enzyme-linked immunosorbent assay (ELISA) according to the manufacturer’s instructions [Human IL-6 ELISA Kit (E-EL-H0192c), Human IFN-γ ELISA Kit (E-EL-H0108c), Human TNF-α ELISA Kit (E-EL-H0109c), Human LBP ELISA Kit (E-EL-H6108), Elabscience; Human LPS ELISA Kit (CSB-E09945h), CUSABIO], and the serum samples were processed in duplicate. The integrity of serum samples was checked by an internal control. The calibration curve for each cytokine was calculated using a four-parameter logistic curve fit regression. Intraassay coefficient variation was consistently <10% for all cytokines.

### 2.3 DNA extraction and 16S ribosomal RNA gene sequencing

Microbial DNA was isolated from ear canal swab samples using the cetyltrimethylammonium bromide (CTAB) and sodium dodecyl sulfate (SDS) (CTAB/SDS) method according to the manufacturer’s instructions. DNA integrity and concentration were monitored *via* 1% agarose gel electrophoresis. The V3-V4 hypervariable regions of the 16S rRNA gene were PCR amplified with specific primers (341F, 5′-CCTACGGGRSGCAGCAG-3′; 806R, 5 ′-GGACTACVVGGGTATCTAATC-3′) and barcodes. The PCR reactions consisted of 15 µL of Phusion^®^ High-Fidelity PCR Master Mix (New England Biolabs), 0.2 µM of each primer, 10ng target DNA and reaction conditions were: 98°C for 1 min, followed by 30 cycles of 98°C for 10 s, 50°C for 30 s, and 72°C for 30 s, and a final 5 min extension at 72°C. Purified DNA was sequenced using a NovaSeq platform and 250 bp paired-end reads were generated. Denoise was performed with DADA2 or deblur module in the QIIME2 software (version QIIME2-202006) to obtain initial Amplicon Sequence Variants (ASVs). Species annotation was performed using QIIME2 software based on the Silva Database. Alpha diversity and beta diversity were calculated in QIIME2 and visualized using the ggplot2 package in the R program (http://www.R-project.org). The 16S rRNA gene amplicon sequencing data from ear canal swab samples were obtained from Beijing Novogene Biotechnology Co., Ltd, China.

### 2.4 Metagenomic sequencing

The metagenomic shotgun sequencing was performed on the Illumina platform at Beijing Novogene Biotechnology Corporation. Raw data generated from shotgun metagenome sequencing were quality filtered and trimmed to remove reads with low-quality bases and potential human contaminants were removed using Bowtie2 (version 2.2.4). Then, MEGAHIT software (v1.0.4-beta) was used for assembly analysis of clean data. Metagenomics taxonomical annotation was performed using DIAMOND (V0.9.9.110), using the NCBI NR database to align unigenes to bacteria, fungi, archaea and viruses sequences. The least common ancestors (LCA) algorithm of MEGAN software was applied to the system classification, the abundance of each sample at each taxonomy (kingdom, phylum, class, order, family, genus, or species) and the corresponding gene abundance tables are acquired. All samples were compared and analyzed by relative abundance. To obtain gene function annotations, DIAMOND software (v0.9.9.110) was used to blast the unigenes against the functional databases (eggNOG, CAZy, and KEGG). According to the alignment results, the relative abundance at different functional levels is calculated.

### 2.5 Statistical analyses

The clinical data were analyzed by the Graph Pad Prism 8.0 software (Graph Pad software, lnc., San Diego, CA, USA). The data consistent with normal distribution were expressed as means ± standard deviation (SD). Student’s t-test was used to determine the significance of differences between the two groups, the nonparametric Mann-Whitney U test was compared when the data did not satisfy a normal distribution. The significance of differences in microbial composition between groups was evaluated using a permutational multivariate analysis of variance (PERMANOVA) test with the adonis function in the vegan R package. The principal coordinate analysis (PCoA) plots were generated using ggplot2 in R version 4.0.2 (http://www.R-project.org/). Linear discriminant analysis effect size (LEfSe), was used to identify taxonomic and functional features which are differentially abundant between groups. Spearman’s rank correlation was used to analyze the correlation between clinical parameters and microbiome features. Microbial markers were identified by ten-fold cross-validation of a random forest model. The diagnostic performance of the optimum biomarker was evaluated using receiver operating characteristic (ROC) curves and the area under the ROC curve (AUC). *P* < 0.05 were considered to be statistically significant.

## 3 Results

### 3.1 Basic characteristics of participants

The demographic and clinical characteristics of the individuals who participated in this study are summarized in **Table 1**. A total of 47 GAD patients during periods of stable disease and 33 HC were assessed. The participants in the two groups exhibited similar age, gender, and BMI distributions. Consistently with previous studies (26), we observed that the GAD patients were predominantly females (61.7%). Each participant was analyzed the severity of anxiety symptoms using the Self-Rating Anxiety Scale (SAS) (27) and Hamilton Anxiety Rating Scale (HAMA) (28). The Pittsburgh Sleep Quality Index (PSQI) (29), Composite Autonomic Symptom Score 31 (COMPASS 31) (30), Gastrointestinal Symptom Rating Scale (GSRS) (31), Fatigue Severity Scale (FSS) (32), Dizziness Handicap Inventory (DHI) (33) were used to evaluate the other comorbid symptoms. Clinical details of the patient cohort are summarized in **Table 1**. We found that 68.1% of the GAD patients have higher DHI scores and comorbid with dizziness (**Table 1**).

**Table 1:** Clinical characteristics of the enrolled participants.

Clinical characteristics	GAD group (n=47)	HC group (n=33)	*P*-value
Female sex (n, %)	29 (61.7%)	20 (60.6%)	NA
Age (years)	49.38 ± 7.59	48.84 ± 7.93	0.995
BMI (kg/m^2)^	23.14 ± 2.70	23.42 ± 2.83	0.682
Duration (years)	4.57 ± 1.70	NA	NA
HAMA	15.34 ± 5.77	4.03 ± 3.68	<0.0001
SAS	64.82 ± 7.52	21.85 ± 10.21	<0.0001
COMPASS 31	23.06 ± 11.94	9.33 ± 5.05	<0.0001
GSRS	6.06 ± 3.35	1.21 ± 1.26	0.434
DHI	30.42 ± 26.25	0.60 ± 1.63	<0.0001
PSQI	12.73 ± 6.10	5.36 ± 3.54	<0.0001
FSS	29.17 ± 11.80	16.58 ± 7.96	<0.0001

Data are shown as the mean ± SD. BMI, body mass index; HAMA, Hamilton Anxiety Scale; SAS, the self-rating anxiety scale; COMPASS 31, Composite Autonomic Symptom Score 31; GSRS, Gastrointestinal Symptom Rating Scale; DHI, Dizziness Handicap Inventory; PSQI, The Pittsburgh sleep quality index; FSS, Fatigue Severity Scale; NA, not applicable.

### 3.2 Substantial alterations in ear canal microbiome composition

**Figure 1:**
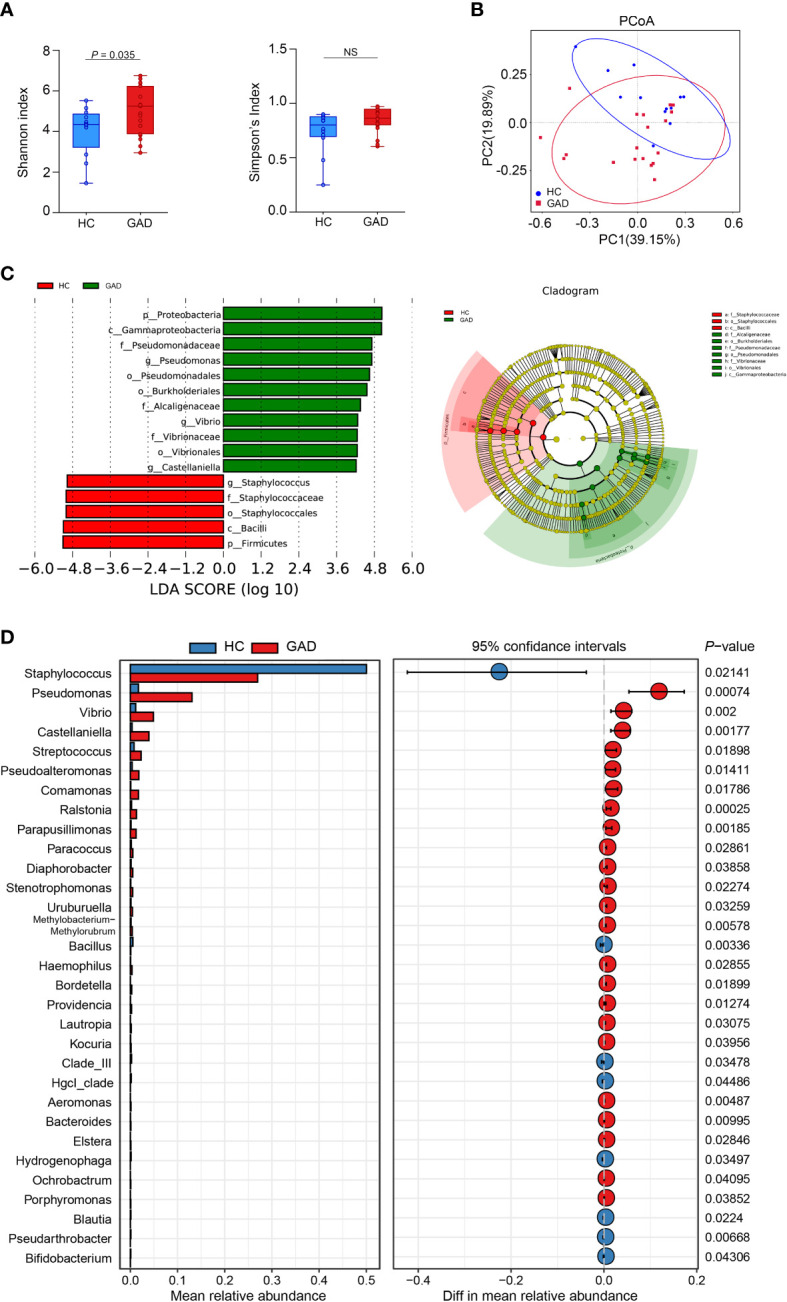
Alteration of ear canal microbiota in GAD patients using 16S rRNA gene amplicon sequencing. **(A)** Boxplots of α-diversity as measured by Shannon diversity and Simpson diversity. Elements show the median, minimum and maximum values. **(B)** Principal coordinate analysis of all samples based on weighted UniFrac distance. *P* values of beta diversity based on weighted UniFrac distance were calculated with permutational multivariate analysis of variance analysis (PERMANOVA). Circles show 95% confidence intervals. **(C)** Linear discriminant analysis (LDA) effect size (LEfSe) analysis revealed significant bacterial differences in ear canal microbiota between the GAD (positive score) and HC groups (negative score). LDA scores (log10) > 4 and *P* < 0.05 are shown. These analyses revealed the most differentially abundant taxa at the level of bacterial phylum (p), class (c), order (o), family (f), and genus (g). **(D)** Statistical analysis of the ear canal microbiota in GAD and HC groups by Mann-Whitney U test. ^NS^ *P* ≥ 0.05.

To investigate the ear canal microbiota community in GAD patients, we initially undertook 16S rRNA gene sequencing on a total of 31 ear canal swab samples from 19 GAD patients and 12 HC. To assess the differences of bacterial diversity between groups, sequences were aligned for α-diversity. The Simpson’s Index of diversity showed no significant differences between groups, while the Shannon index was significantly higher in the GAD group (**Figure 1A**, *P* = 0.035). To display microbiome space between samples, beta diversity was calculated based on Bray-Curtis distances, visualized by principal coordinates analysis (PCoA) plot. The results showed a significantly different distribution between groups using permutational multivariate analysis of variance (PERMANOVA) analysis (**Figure 1B**, *P* < 0.05). To explore the most differentially abundant taxa in GAD and HC groups, we performed linear discriminant analysis coupled with effect size analysis (LEfSe) algorithms on ear canal microbiota composition based on the 16S rRNA gene sequencing. 16 bacterial taxa showed different relative abundances between GAD and HC groups (linear discriminant analysis (LDA) score > 4.0, *P* < 0.05). The genera significantly increased in abundance in the GAD group including *Pseudomonas*, *Vibrio*, *Castellaniella*, and *Pseudoalteromonas*, all common skin bacteria as well as occurring in the gut, from the phylum *Proteobacteria*. The genera decreased in GAD group including *Staphylococcus* and *Bacillus*, from the phylum *Firmicutes*. We further identified *Pseudomonas* and *Staphylococcus* in the genus level as the main microbiota in the GAD and HC groups, respectively (**Figures 1C, D**).

**Figure 2:**
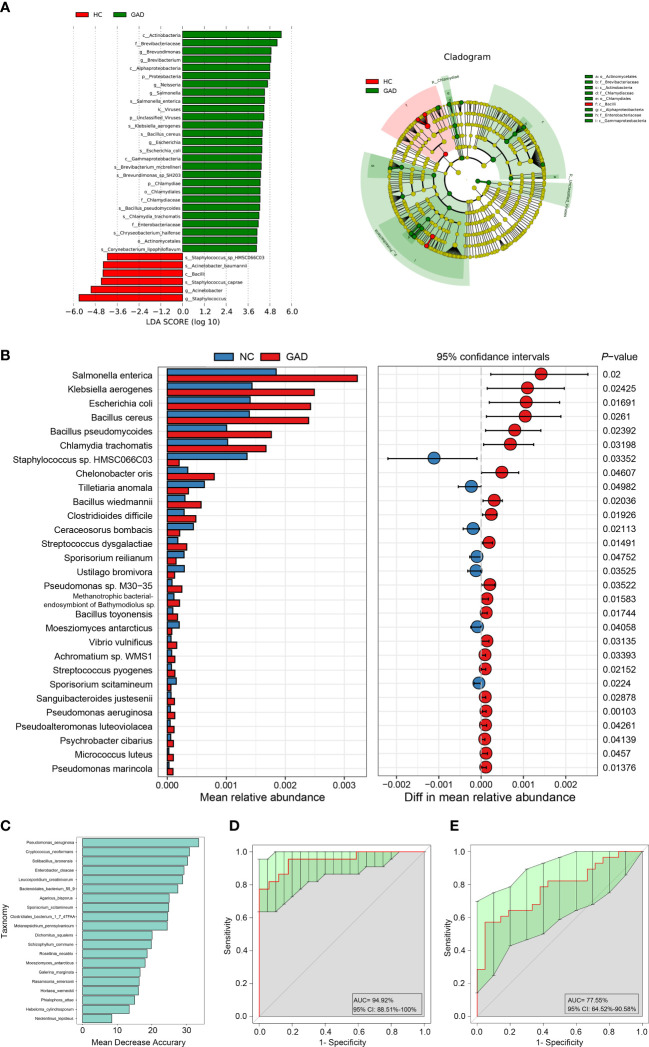
Metagenomic sequencing-based exploration of ear canal microbiota in GAD patients. **(A)** LEfSe analysis revealed significant bacterial differences in ear canal microbiota between the GAD (positive score) and HC groups (negative score). LDA scores (log10) > 4 and *P* < 0.05 are shown. These analyses revealed the most differentially abundant taxa at the level of bacterial phylum (p), class (c), order (o), family (f), genus (g), and species(s). **(B)** Statistical analysis of the ear canal microbiota in GAD and HC groups by Mann-Whitney U test. **(C)** Top 20 differentially abundant genera markers were selected as the optimal marker set based on random forest between 28 GAD patients and 21 HC. The x-axis presents the mean decrease accuracy to each marker, which indicates the contribution to the accuracy of the model. **(D)** Receiver operating characteristic curve (ROC) describing the prediction accuracy of model calculated using a 10-fold cross-validation based on top 20 differentially abundant genera markers. Error bars denote 95% confidence interval for AUC value. **(E)** ROC describing the prediction accuracy of model calculated based on the relative abundance of *Pseudomonas aeruginosa*. Error bars denote 95% confidence interval for AUC value.

To further identify species in the ear canal microbiota, metagenomic sequencing with species-level taxonomic resolution was performed (GAD, n = 28; HC, n = 21). The findings presented that genus *Staphylococcus* was the key microbiota in the HC group, whereas genus *Brevundimonas* was the dominant microbiota in the GAD group using LEfSe analysis (**Figure 2A**, LDA score > 4.0, P < 0.05). Then, species-level microbial markers were also characterized between GAD patients and HC by Welch’s T test. Compared to HC group, bacterial species of *Salmonella enterica*, *Klebsiella aerogenes, Escherichia coli*, *Bacillus cereus*, *Streptococcus dysgalactiae*, *Pseudomonas* sp. M30-35 and *Pseudomonas aeruginosa* were significantly increased in GAD group. Species of *Staphylococcus* sp. HMSC066C03 and *Staphylococcus caprae* were consistently the dominant populations in HC group, detected by both 16S rRNA gene and metagenomic sequencing (**Figure 2B**). These results demonstrated profound imbalances in the composition and inter-species relationship in the ear canal microbiome of GAD patients as compared to those of HC. We next assessed the potential value of using the ear canal microbiota as biomarkers. We performed 10-fold cross-validation to estimate the prediction accuracy, dividing the disease cohort into 90% training sets and a 10% discovery set. A selection of the top 20 taxonomies consist *Pseudomonas aeruginosa*, with the largest effect size in the prediction model was tested, resulting in an AUC mean of 0.95 (**Figures 2C, D**; 95% CI:0.88–1.00). *Pseudomonas aeruginosa* was frequently selected as the most important species, singularly predicting GAD with an AUC of 0.78 (**Figure 2E**, 95% CI:0.65–0.90). In general, our results suggested that the ear canal microbiota is considered one of the most promising biomarkers for GAD diagnosis.

### 3.3 Changes in the ear canal microbiome linked to the increased systemic inflammation in GAD patients

**Figure 3:**
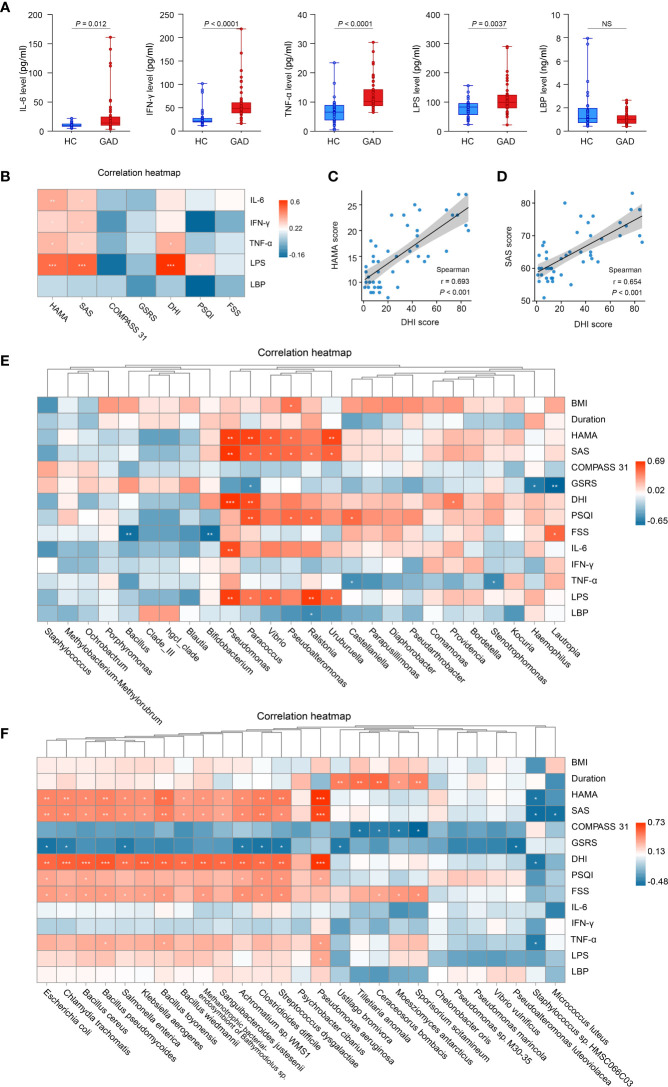
Altered systemic inflammatory cytokines in GAD patients and the correlations between clinical symptoms and abundances of taxonomic differences of ear canal microbiota. **(A)** Comparison of systemic inflammatory biomarker levels (IL-6, IFN-γ, TNF-α, LPS, and LBP) from serum samples in GAD and HC groups. Results are expressed as median and range. **(B)** Heatmap showing Spearman’s correlation between clinical parameters and inflammatory cytokines between groups. Red denotes positive correlation and blue denotes negative correlation. **(C)** Spearman’s correlation between the DHI score and HAMA score. **(D)** Spearman’s correlation between the DHI score and SAS score. **(E)** Heatmap showing Spearman’s correlation between clinical parameters and differentially abundant genus between groups. Red denotes positive correlation and blue denotes negative correlation. **(F)** Heatmap showing Spearman’s correlation between clinical parameters and differentially abundant species between groups. Red denotes positive correlation and blue denotes negative correlation.*P < 0.05, **P < 0.01, and ***P < 0.001; IL-6, interleukin-6; TNF-α, tumor necrosis factor-α; IFN-γ, interferon-γ; LPS, lipopolysaccharide; LBP, lipopolysaccharide-binding protein; HAMA, Hamilton Anxiety Scale; SAS, the self-rating anxiety scale; COMPASS 31, Composite Autonomic Symptom Score 31; GSRS, Gastrointestinal Symptom Rating Scale; DHI, Dizziness Handicap Inventory; PSQI, The Pittsburgh sleep quality index; FSS, Fatigue Severity Scale. ^NS^P≥0.05.

To confirm the role of systemic inflammation in GAD, we first measured the levels of pro-inflammatory cytokines and inflammatory agonists (LPS) in serum from GAD patients and HC. Higher levels of IL-6, IFN-γ, TNF-α and LPS were detected in GAD patients compared to HC (**Figure 3A**). However, the serum levels of LBP, an acute phase protein critical to innate immune responses to Gram-negative infections, were reduced in GAD patients compared to HC (**Figure 3A**). Furthermore, we found that the HAMA and SAS scores were positively correlated with DHI scores (**Figure 3C**, r = 0.693, *P* < 0.001; **Figure 3D**, r = 0.654, *P* < 0.001). In addition, the levels of IL-6, IFN-γ, TNF-α and LPS levels were found to have a significantly positive correlation with the HAMA and SAS scores. TNF-α and LPS levels were also significantly positively associated with DHI scores (**Figure 3B**).

Next, we tried to determine whether the changes in the ear canal microbiome of GAD correlated with the specific cytokine responses and the severity of GAD. Spearman correlations were applied to detect the differentially abundant genus and species of GAD and HC groups based on 16S rRNA gene sequencing and metagenomic sequencing, respectively. We found that the genus of *Pseudomonas*, *Paracoccus*, *Vibrio*, and *Uruburuella* were significantly positive associated with serum LPS levels and HAMA, SAS scores. The relative abundance of *Pseudomonas aeruginosa* were significantly positive associated with TNF-α and LPS levels. Moreover, *Pseudomonas* and *Paracoccus* were significantly positive associated with DHI score. Meanwhile, relative abundance of *Bacillus* and *Bifidobacterium* were observed significantly negative associated with FSS score (**Figure 3E**). Furthermore, we also found that the increased species in GAD group, including *Pseudomonas aeruginosa, Streptococcus dysgalactiae*, *Clostridioides difficile*, *Bacillus pseudomycoides*, *Chalmydia trachomatis*, *Bacillus toyonensis*, and *Escherichia coli*, etc., were significantly positive associated with DHI, SAS, FSS and HAMA scores, and inversely correlated with GSRS score. Additionally, negative correlations were observed between *Staphylococcus* sp. HMSC066C03 and disease severity (**Figure 3F**). These results suggest that the altered ear canal microbiota in GAD patients is associated with systemic inflammatory responses that contribute to GAD development.

### 3.4 Functional alterations in the GAD ear canal microbiome

**Figure 4:**
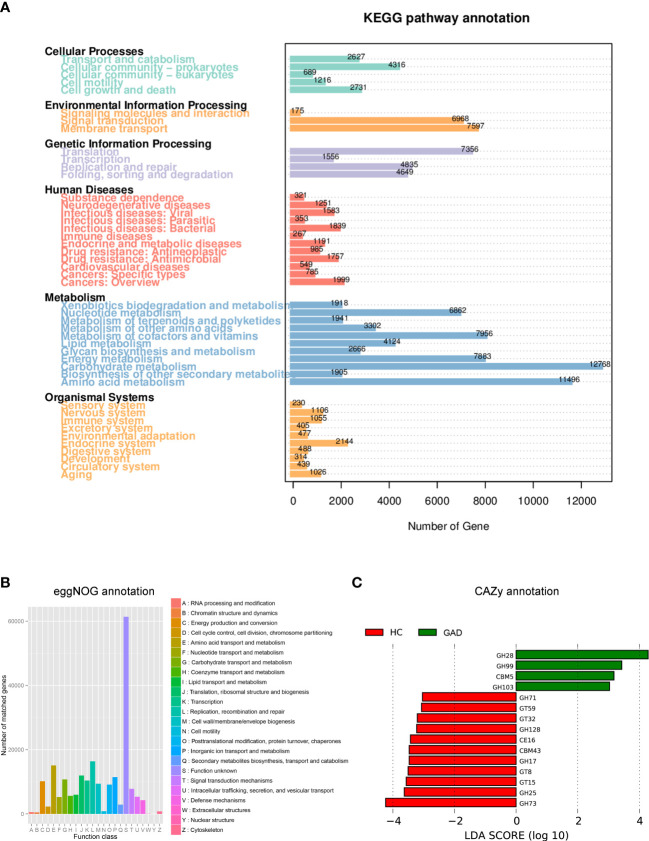
Functional predictions for the ear canal microbiome of patients with GAD. **(A)** Different abundance levels of genes annotated according to functional classification based on Kyoto Encyclopedia of Genes and Genomes (KEGG) databases. **(B)** Different abundance levels of genes annotated according to functional classification based on evolutionary genealogy of genes: non-supervised orthologous groups (eggNOG) database. **(C)** LEfSe analysis revealed significant predicted function based on alignment with Carbohydrate-Active enzymes (CAZy) database between the GAD (positive score) and HC groups (negative score). LDA scores (log10) > 3 and *P* < 0.05 are shown.

Dysbiosis of the microbiota can alter metabolic pathways, and metabolic dysfunction can change microbiota composition (34, 35). To study the functional and metabolic changes of the ear canal microbial communities, metagenomic reads were annotated with predicted function based on alignment against available databases [Kyoto Encyclopedia of Genes and Genomes (KEGG), Carbohydrate-Active enzymes Database (CAZy), and evolutionary genealogy of genes: non-supervised orthologous groups (eggNOG)], for a gene-centric analysis of unassembled metagenomes. As a result, the most abundant KEGG orthologue and the eggNOG annotations were analyzed in all samples (GAD, n = 28; HC, n =21). The most abundant KEGG orthologues were involved in metabolism, including the carbohydrate metabolism, amino acid metabolism, metabolism of cofactors and vitamins, and energy metabolism (**Figure 4A**). According to the eggNOG annotations, the most abundant NOGs were amino acid transport and metabolism; replication, recombination and repair (**Figure 4B**). However, there was no significant difference in overall predicted functional capacity between GAD and HC groups in a global comparison of all annotated domains. In CAZy-enrichment analysis, several annotated functions were distinct between the two groups. LEfSe analysis revealed that the Glycoside Hydrolases family 28 (GH28) was the key term increased in GAD group, while GH73, GH25, Glycosyl Transferases (GT15), and GT8 were overrepresented in HC group (**Figure 4C**, LDA score > 3.0, *P* < 0.05).

## 4 Discussion

Increasing evidence has revealed the important role of microbiota-gut-brain axis in anxiety (15, 36, 37). However, the ear canal microbiota profile in anxiety patients has not been characterized previously. Here, we present the analysis of the ear canal microbiome in patients with GAD. Our results demonstrated that the ear canal microbiome of GAD patients is significantly different from that of HC. More importantly, the alteration of microbiota composition highly correlated with the elevated serum concentrations of TNF-α and LPS, as well as the anxiety symptom severity in patients with GAD. Additionally, animal studies showed exposure to pathogenic bacteria in the gastrointestinal tract can increase anxiety-like behavior through producing exotoxins and promoting favoring inflammation conditions (14, 38). Taken together, we infer that specific genera of ear canal microbiota may associate with the risk and severity of disease *via* upregulating inflammasome signaling modulate pathways.

Dizziness is one of the most prevalent somatic symptoms in GAD (39, 40). Our findings showed the increased relative abundance of *Pseudomonas aeruginosa* in the ear microbiome of GAD patients compared to that of HC, and the strongest correlations between the relative abundance of *Pseudomonas aeruginosa* and DHI scores. We hypothesized that changes in ear canal microbiota of GAD might contribute to the vestibular deficit. Previous studies indicated that vestibular systems reciprocally connected with a myriad of anxiety-related brain areas, including the dorsal raphe nucleus, locus coeruleus, parabrachial nucleus and hypothalamus (41, 42). *Pseudomonas aeruginosa* is a common opportunistic pathogen and mainly affects immunocompromised individuals (43). Increased abundance of *Pseudomonas aeruginosa* has been observed in chronic suppurative otitis media patients (44, 45). Moreover, almost 70% of the chronic suppurative otitis media patients had vestibular symptoms (46). Overall, our findings suggest that the increased relative abundance of *Pseudomonas aeruginosa* in the ear microbiome may induce dizziness in GAD patients.

*Pseudomonas aeruginosa* is an aerobic gram-negative bacterium belonging to the *gamma*-*Proteobacteria*, widely distributed in the environment (47). LPS is a major component of the outer membrane of gram-negative bacteria and frequently plays an important role in activating inflammatory responses (48). The present study showed that the level of serum LPS was increased and positively correlated with the abundance of *Pseudomonas aeruginosa* in GAD patients. Previous studies also reported that *Pseudomonas aeruginosa* induces macrophages to release various proinflammatory cytokines and chemokines through activation of the TLR4/MyD88/NF-κB signaling pathway (49, 50). Our results provide evidence for the association between systemic inflammatory response and ear canal dysbiosis. Whether the antibiotic administration against *Pseudomonas aeruginosa* to improve the inflammatory responses due to GAD needs to be further investigated in future studies.

Increasing evidence has demonstrated that GAD is heavily influenced by stress pathways such as the HPA axis. Dysbiosis may be one pathway through which stress impacts the HPA axis. Dysregulation of several bacteria has been hypothesized to be associated with psychiatric illnesses (51, 52). Our study identified several obviously altered bacteria producing neurotransmitters in the ear microbiome, such as increased *Escherichia coli* and *Bacillus* for norepinephrine and dopamine, decreased *Bifidobacterium* for gamma-aminobutyric acid (GABA) (51). These findings provide clues for the view that altered ear canal microbiome may influence anxiety behavior through the HPA axis, although this would need animal experimental validation.

This study revealed that ear canal microbiota may play an essential env.1ironmental role in the pathogenesis of the GAD. However, there are several limitations of the exploratory study that should be mentioned and discussed. First, recognized variations in ear canal microbiome profiles between individuals and confounders such as living environment likely limited our ability to detect additional significant taxonomic biomarkers for GAD. A design incorporating such repeated sampling of the same individual would also help overcome the problem of inter-individual variation. Second, the participants of this study were all from a single center with a small sample size. Large-sample and multicenter studies are still needed to confirm our preliminary results in the future. Finally, we only demonstrated the alteration of the ear canal microbiome in our study, microbiomes from other body sites, such as the gut, the skin, the oral cavities, or the bladder, should be also considered. Alteration in the microbiota in other organs was also able to induce changes in inflammatory cytokines that contribute to the pathogenesis of GAD. Despite these limitations, a discriminatory signal is present in both the 16S rDNA and metagenomic datasets supporting the ear canal as a potential source of disease biomarkers in GAD. These candidates should be further evaluated for their mechanistic and causal involvement in GAD using established animal models.

## Data availability statement

The datasets presented in this study can be found in the NCBI SRA BioProject repositories, accession number PRJNA930499; PRJNA929308.

## Ethics statement

The studies involving human participants were reviewed and approved by the Ethics Committee of The First Affiliated Hospital of Zhengzhou University. The patients/participants provided their written informed consent to participate in this study.

## Author contributions

XD conceived and designed the experiments; XD and XW coordinated the whole project; RF, QZ and QL were responsible for the initial assessing and diagnosing patients; CQ, and YZ were responsible for assessing, documenting their patients’ health information; JW recorded and confirmed the data; DL, RZ, HT, HL, YC, and YF collected the samples of participants; XW and XD provided statistical analysis and technical support; RF, QZ, XD, and XW participated in final data analysis and interpretation; RF, XD, and XW did most of the writing with input from other authors; all of the authors discussed the results and commented on the manuscript. All authors contributed to the article and approved the submitted version.
